# Molecular guided therapy leading to exceptional response in relapsed osteosarcoma

**DOI:** 10.3389/fphar.2025.1719832

**Published:** 2026-01-05

**Authors:** Elizabeth Wert, Leah Menachery, Jeremy Hengst, Tarlan Arjmandi, Abhinav B. Nagulapally, Divya Gandra, Valerie Brown, Giselle Saulnier Sholler, Smita Dandekar

**Affiliations:** 1 The University of North Carolina at Chapel Hill, Chapel Hill, NC, United States; 2 Department of Pediatrics, Division of Hematology and Oncology, Penn State Health Golisano Children’s Hospital and Penn State College of Medicine, Hershey, PA, United States

**Keywords:** genomics, molecular tumor board, osteosarcoma, precision medicine, targeted therapy

## Abstract

Osteosarcoma is the most common type of primary malignant bone tumor in children, adolescents and young adults and remains a significant clinical challenge, especially in the context of metastatic disease. Here we report the case of a 9-year-old female with refractory metastatic osteoblastic osteosarcoma with disease progression in the lungs following neoadjuvant chemotherapy, local control surgery with limb salvage and further aggressive chemotherapy. She was then enrolled on a Molecularly Guided Therapy Clinical Trial (NMTRC009) utilizing genomic analysis to identify novel treatment options. Whole exome sequencing (WES) and RNA-Seq were performed on each patient’s tumor to identify genomic aberrations when referenced to normal tissue. WES of the tumor identified no targetable mutations. RNA transcriptome sequencing of the subject’s tumor showed overexpression of SLC29A11 (Z-score = 3.3) indicating sensitivity to gemcitabine as well as activation of the biological pathways mTOR, CSF1R, EPHA2, SLC29A1, suggesting possible beneficial treatment with a combination of everolimus, gemcitabine, doxycycline and dasatinib. Cell viability assays on the subject derived cell line SL00339 showed minimal effects of single agent treatments but a significant decrease in cell viability with combination therapies. Western blot analysis of cells treated with drugs alone and in combination showed an increase in apoptosis and decrease in pmTOR and pAKT. The subject responded to the novel drug combination, continuing medications for 5 years with some modifications, and remained on everolimus alone for an additional 4 years with a complete response, no serious adverse events, and excellent quality of life. In conclusion, Molecular Guided Therapy with tumor board recommendations resulted in a novel therapeutic approach leading to long term survival which correlated to response *in vitro*.

## Introduction

Osteosarcoma is the most common type of primary malignant bone tumor in children, adolescents and young adults accounting for approximately 5% of all childhood tumors. Patients who are non-metastatic at diagnosis have a survival rate of approximately 76%, while those who are metastatic at diagnosis have a survival rate of 24% (Society, 2023; Phillip and Pizzo, 1989; Database NCIS, 2022). The survival rate once relapsed or refractory to treatment is 20% (Spraker-Perlman et al., 2019). Survival prognosis after recurrence is associated with age at the time of relapse, extent of disease at diagnosis (localized vs. metastatic), site(s) of relapse, and time to relapse. Age at first relapse <18 years, localized disease at diagnosis, relapse >2 years post diagnosis, and not having a combination relapse (bone/lung) are favorable prognostic factors at relapse (Spraker-Perlman et al., 2019). Despite a multi-disciplinary treatment approach of neo-adjuvant chemotherapy, radiation and/or surgery, including limb salvage for tumors involving the extremities, not much progress has been made in the past 3.5 decades in improving the overall survival rates for osteosarcoma (Isakoff et al., 2015; Jaffe, 2009). Successfully treating patients who develop pulmonary metastases after receiving multimodality treatment is particularly challenging.

Understanding the underlying biology and molecular alterations in these cancers is incomplete. Unlike many sarcomas which are characterized by specific chromosome translocations, osteosarcoma exhibits significant genomic heterogeneity and instability. The genomic heterogeneity is characterized by high levels of structural variations, mutations, and copy number alterations (Schott et al., 2020). While these alterations contribute to the complexity of osteosarcoma genomes, few have been directly linked to clinical outcomes, making it challenging to identify targeted therapies (Meltzer and Helman, 2021; Morrow and Khanna, 2015).

Collaborative efforts are greatly needed to understand the biology of osteosarcoma and to develop and use preclinical models to test novel agents to improve outcomes for patients. These would allow for improved targeted therapy options for patients with metastatic osteosarcoma. More complete genomic sequencing and analysis may lead to the identification of novel therapies. One such approach of personalized therapy directed towards tumor biology through genomic sequencing is described here, which resulted in an exceptional patient response.

## Case description

In September 2014, a 9-year-old female presented with a 4-month history of pain above the right knee, difficulty bearing weight on the right leg, fatigue and a 7 lb. Weight loss. On exam, there was swelling at and above the knee, with a palpable mass and decreased range of motion. After conservative management for a few months, an Xray of the knee revealed a permeative osseous lesion in the right distal femoral metaphysis, with a pathological fracture through the distal right femoral lesion. An MRI of the right lower extremity confirmed an aggressive tumor arising from the right femoral metaphysis with an expansile soft tissue mass, extending into the knee joint. CT showed multiple pulmonary nodules, concerning for metastatic disease. Biopsy of the mass confirmed the diagnosis of osteoblastic osteosarcoma with extraosseous extension. She received neoadjuvant therapy with cisplatin, doxorubicin, and high-dose methotrexate for 10 weeks followed by a 5-day cycle of ifosfamide and etoposide. Imaging after 11 weeks of chemotherapy showed a significant decrease in number and size of the pulmonary nodules with minimal change in size of the tumor involving the right femur.

In January 2015, she underwent local control surgery with a Van Ness Rotationplasty. Negative margins were attained, but tumor necrosis was only 80%, making her a “Poor Responder” by histologic response. She continued adjuvant chemotherapy until a routine Chest CT done 1-month post-surgery showed an increase in the size of a previous right lower lobe lung (RLL) nodule and a new RLL nodule. After one round of high dose ifosfamide and one course of high dose methotrexate, chest CT showed a continued increase in the size of the two lung nodules and a new RLL nodule. She was found to be refractory to current therapy with progression of disease. She had a right thoracoscopy with RLL wedge excision to remove all three right lung nodules. Pathology confirmed metastatic osteosarcoma.

### Enrollment on molecularly guided therapy clinical trial

In May 2015, the patient was enrolled on Molecular Guided Therapy NMTRC009 study “Feasibility Trial Using Molecular Guided Therapy for the Treatment of Patients with Relapsed and Refractory Childhood Cancer” after obtaining written informed consent for study. This study was prospectively registered on ClinicalTrials.gov with identifier: NCT02162732 and was conducted under an FDA exemption (no significant risk). The tumor was sent for DNA whole exome sequencing (WES) and RNA transcriptome sequencing (RNA-Seq), and the results of the genomic analyses were discussed in a Molecular Tumor Board (MTB) where a precision medicine therapy regimen was designed for this patient. Patient safety was evaluated by monitoring adverse events, and response was determined by radiological examination with serial CTs of the chest.

### Genomic sequencing

**FIGURE 1:**
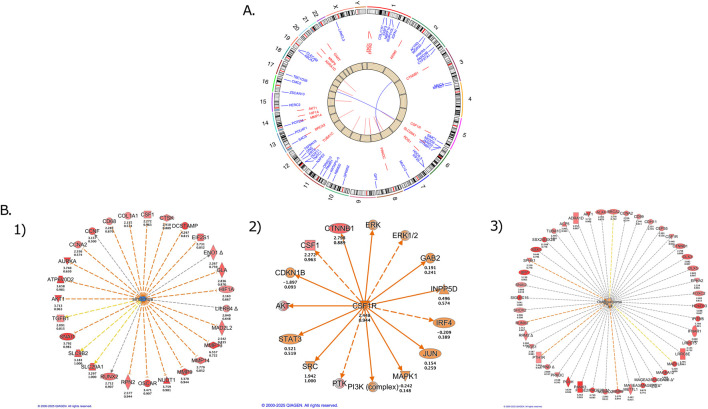
Genomic Analysis of the Osteosarcoma Patient **(A)** Circos plot depicting DNA chromosomes withgene mutations in blue, RNA-Seq gene overexpression in red, copy number alteration in tan, and internal fusions/translocations with lines in the center **(B)** Differentially expressed genes were identified and based on Z-Scores and relevant pathways were generated using Ingenuity Pathway Analysis (IPA) was used to identify significant changes in the Pathways/Regulators/Diseases. 1) Sirolimus is indicated as a targeted therapeutic agent due to the elevated expression of downstream genes. 2) CSF1R is highly activated and correlates with elevated expression of downstream genes. **(C)** Osteosarcoma as a disease has numerous highly activated downstream genes with elevated expression.

The pathology of the tumor showed 100% cell viability with numerous atypical mitoses. WES and RNA-Seq were performed and analyzed as per study NMTRC009 (Sholler et al., 2024) for presentation at the Molecular Tumor Board. RNA expression was reported by Z-score relative to normal tissue (Sholler et al., 2024) and by Cancer Reference Control (CRC) percentile relative to other pediatric cancers (Sholler et al., 2024). Figure 1A shows the Circos Plot with DNA mutations as well as the RNA expression changes identified. Differentially expressed genes were identified and based on Z-Scores and relevant pathways were generated using QIAGEN IPA (QIAGEN Inc., https://digitalinsights.qiagen.com/IPA). As shown in Figure 1B(3), osteosarcoma is regarded as a tumor type with highly activated signaling as reflected by upregulation of numerous signaling pathways. IPA identified mTOR inhibitors (Figure 1B(1)) as targeted agents with potential therapeutic efficacy (Z-score = 3.7). IPA also identified CSRF1 as a highly overexpressed signaling hub (Z-score = 3.3) with links to PI3K/AKT and STAT3 pathways (Figure 1B(2)).

### Cell line generation

The subject’s cancer cell line, SL00339, was generated from the tumor. The tumor was collected fresh at surgery, minced and placed in culture media for 15–20 min in a 10 cm dish; following incubation, all media and tumor pieces were transferred to a T25 flask and cultured following standard practices and maintained in MEMα+ with 10% FBS, 100 U/mL Penicillin, and 100 μg/mL Streptomycin.

### Cell viability assays

**FIGURE 2:**
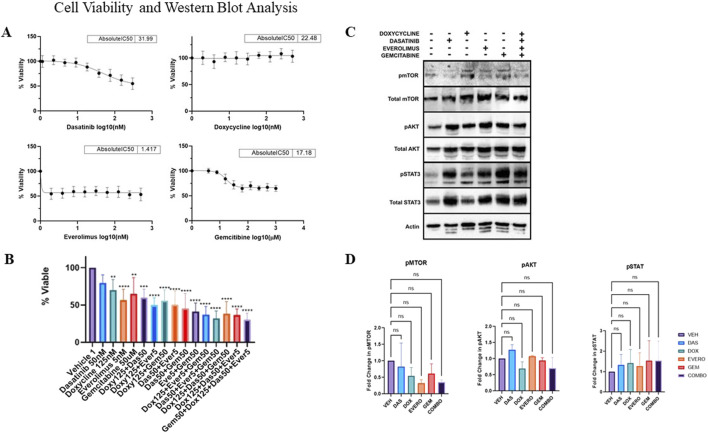
**(A)** Osteosarcoma patient cells were treated with dasatinib, doxycycline, gemcitabine, and everolimus for 72 h. Cell viability was measured using CellTiter-Glo to determine IC50 values for each drug. **(B)** Cells were treated with drugs alone and in combination for 72 h at the indicated concentrations. Cell viability was evaluated with CellTiter-Glo after 72 h of treatment (**p-value <0.01, ***p-value <0.0002 **** p-value < 0.0001). **(C)** Representative western blots of cells treated with each drug at the concentrations indicated in **(B)**. Cells were treated with doxycycline, dasatinib and everolimus for 48 h or gemcitabine for 24 h. Cell lysates were prepared and examined by western blot analysis with the indicated antibodies for pAKT/AKT, pmTOR/mTOR, pSTAT3/STAT3, and Beta Actin. Western blots from three individual experiments (n = 3) were quantified by densitometric analysis using ImageJ. **(D)** Statistical significance of the relative fold change for each treatment was determined using GraphPad Prism (ns = not significant).

Patient derived osteosarcoma cells were seeded at 5,000 cells per well in 96-well plates. Plates were treated with single agents at decreasing concentrations starting from their respective C_max_ (C_max_ Values: doxycycline 500 nM, dasatinib 300 nM, gemcitabine 100 uM, everolimus 500 nM). After 72 h, cell viability was determined using CellTiter-Glo Luminescent reagent. As shown in Figure 2A, IC50 values were determined using GraphPad Prism. Gemcitabine and dasatinib showed the greatest single-agent activity, while doxycycline had minimal effect. Everolimus produced an initial decline in viability, which plateaued. All experiments were performed in triplicate.

As shown in Figure 2B, patient cells were treated with agents singly and in combinations. The chosen concentrations were based on single agent IC50 curve results generated above (doxycycline 125 nM, dasatinib 50 nM, gemcitabine 50 uM, everolimus 5 nM). Single agents induced limited cell death compared to combination treatments. In contrast, drug combinations significantly reduced cell viability, with two-drug combinations reducing viability more than single agents, and triple-drug combinations showing greater effect. The most effective drug combination was gemcitabine, doxycycline, dasatinib, and everolimus demonstrating a statistically significant decrease relative to single agents (p value < 0.0001).

### Western blots targeting specific relevant pathways

Patient derived osteosarcoma cells were plated at 100,000 cells per well overnight in 6 well plates and treated using the drug concentrations employed in Figure 2B. After 48 h, lysates were harvested from each sample and quantified via Bicinchoninic Acid Assay to ensure equal loading. Samples were denatured, reduced, and run on SDS-PAGE, followed by a transfer from the gel to PVDF membranes. Membranes were blocked, probed with primary antibodies, washed, and incubated with secondary antibodies. Protein detection was performed using chemiluminescence imaging. Blots were probed with pAKT, AKT, pmTOR, mTOR, pSTAT3, STAT3, and Beta Actin (Cell Signaling).

Figure 2C shows the effects of the single and combination drug treatments on the AKT, mTOR and STAT3 signaling pathways. We observed that the single and combination treatments had no effect on phosphorylation of STAT3. We also observed a trend toward a decrease in pAKT in response to doxocycline and a decrease in pmTOR by both doxycycline and everolimus. The combination treatments show a similar trend in downregulation of pAKT and pmTOR. However, as shown in Figure 2D, quantitation of the single and combination treatments revealed that the observed changes did not reach statistical significance.

While neither the single agents nor the combination significantly inhibited any of the signaling pathways, decreases in pAKT and pmTOR signaling indicate that the agents were affecting these pathways at the concentration employed. It is possible that with additional timepoints or drug concentrations significance may be reached. Given that the combination significantly reduced cell viability as shown in Figure 2B it is possible that the combined effects of decreased AKT and mTOR signaling could play a role in the reduction in cell viability. These are pro-survival pathways, and their reduction is consistent with the observation of tumor inhibition. However, we cannot exclude the possibility that other signaling pathways were affected by the combination of agents.

### Molecular tumor board decision

**FIGURE 3:**
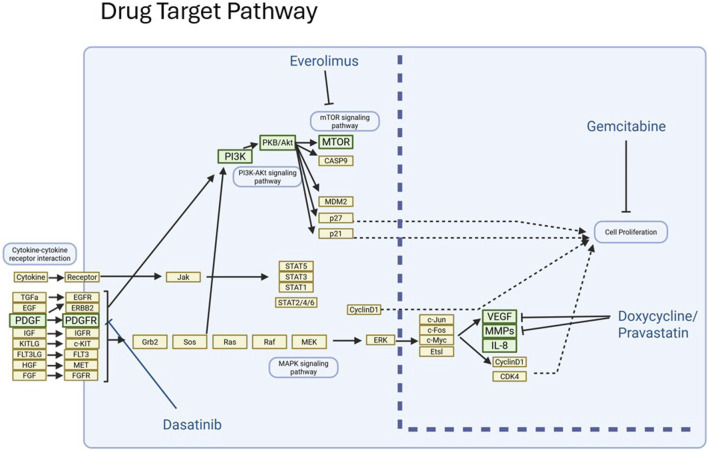
Drug target Pathway.

Based on the genomic analysis of the subject’s tumor, the MTB consisting of oncologists, pharmacists, bioinformaticians, and researchers, discussed the subject’s previous therapy and current condition. Recognizing the cumulative doses of previously administered chemotherapy agents, a treatment regimen was chosen taking into account safety, toxicity profile, and targeted mechanism. The chosen pharmacologic agents included gemcitabine (targeting SLC29A1, ABCC10), everolimus (targeting mTOR), dasatinib (targeting CSF1R, EPHA2, SRC, TEC) and doxycycline (targeting MMP9) as shown in Figure 3. The MTB considered pravastatin (targeting MMP9, MMP14, ICAM) as an alternative agent to doxycycline. Table 1 shows the Molecular Tumor Board (MTB) Treatment Plan.

**TABLE 1:** Molecular tumor board treatment determination.

​	Drug Name	Dose	Route	Schedule	Target/Rationale
1	Dasatinib	38 mg/m^2^/dose	PO	50 mg QD	CSF1R, EPHA2, SRC, TEC Blocks migration and invasion
2	Doxycycline	2 mg/kg/dose	PO	100 mg BID	MMP9 Blocks degradation of bone in the extracellular matrix
3	Everolimus	5 mg/dose	PO	5 mg QD	MTOR, AKT1 Inhibits multiple relevant pathways signaling
4	Gemcitabine	1000 mg/m^2^/dose	IV	Days 1 and 8	SLC29A1, ABCC10 Damages DNA

### Treatment response and adverse events

**FIGURE 4:**
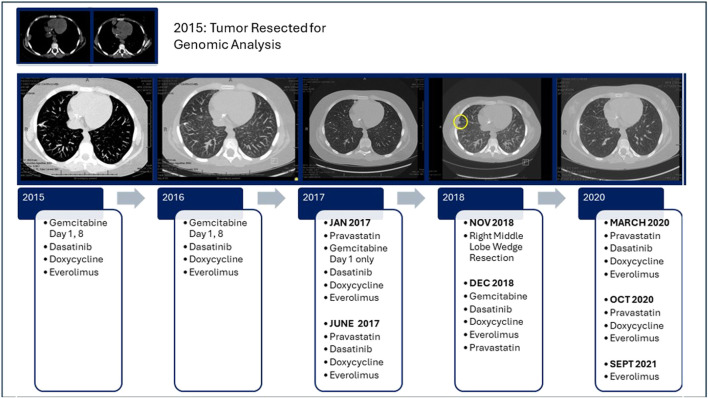
Timeline of treatment and response.

The subject was treated with the MTB Treatment Plan for 12 months until May 2016 due to the finding of 3 new nodules on a Chest CT, Figure 4. Due to the location and the small size of nodules, resection/biopsy was deemed difficult and not performed. The nodules did not grow, and retrospective analysis of imaging showed that they were present on previous CT scans. Treatment was continued. In January 2017, gemcitabine was decreased to be given on Day 1 of each cycle only due to its myelosuppression, and pravastatin was added to the combination of everolimus, dasatinib and doxycycline. In June 2017, Gemcitabine was discontinued completely. In November 2018, a right thoracotomy with right middle lobe wedge resection was performed to remove a new right sided nodule. After surgery, the gemcitabine was restarted until discontinuation in March 2020. Dasatinib was discontinued in October 2020. Subject continued receiving pravastatin and doxycycline until September 2021 and since that time has remained on everolimus alone since September 2021 with surveillance chest imaging showing no active disease now greater than 10 years since enrollment on study. The right lower extremity has shown no evidence of disease recurrence since the original local control Van Ness rotationplasty surgery. The patient tolerated the treatment well without any unexpected significant adverse events (Table 2).

**TABLE 2:** Adverse events.

Adverse Event	SAE	Highest Grade Reported	Relationship to Study Drugs	Actions Taken	Expected
Alanine Aminotransferase Increased (ALT)	No	2	Related to Gemcitabine	None	Yes
Anemia	No	3	Related to Gemcitabine	Required Transfusions	Yes
Aspartate Aminotransferase Increased (AST)	No	2	Related to Gemcitabine	None	Yes
Electrocardiogram QT Corrected Interval Prolonged	No	2	Related to Dasatinib	Study Drug Held; Change in Dose (Dasatinib restarted at same dose but every other day rather than daily)	Yes
Hypertriglyceridemia	No	2	Related to Everolimus	None	Yes
Infusion Related Reaction	No	2	Unrelated to Study Drugs; patient reaction to platelets	Required Therapy	No
Lymphocyte Count Decreased	No	3	Related to Gemcitabine	None	Yes
Muscle Weakness Upper Limb	No	2	Unrelated to Study Drugs	None	No
Neutrophil Count Decreased	No	4	Related to Gemcitabine and Dasatinib	Required Therapy; Study Drugs Held; Change in Dose (decreased Gemcitabine to 75%; then to 56% of original dose)	Yes
Pain - Extremity	No	2	Unrelated to Study Drugs	Required Therapy	No
Platelet Count Decreased	No	4	Related to Gemcitabine and Dasatinib	Required Transfusions; Study Drug Held.	Yes
Vomiting	No	2	Unrelated to Study Drugs	Required Therapy	Yes
White Blood Cell Decreased	No	3	Related to Gemcitabine	Required Therapy; Study Drug Held; Change in Dose (decreased to 56% of original dose; same reduction as listed above)	Yes

## Discussion

Molecularly guided therapy tailored to the patient’s tumor genetic profile offers a novel therapeutic approach, especially for patients that are relapsed or refractory to standard therapy and without options, fulfilling an unmet medical need. In this case study, we report a child with refractory metastatic osteosarcoma, enrolled on a molecularly guided therapy clinical trial. Precision genomics with molecular profiling of the subject’s tumor and discussion in a MTB allowed the creation of a unique treatment plan based on evaluation of genes highly over and under expressed relative to normal tissues and by pathways analysis.

A number of factors were considered in determining the treatment plan for this patient. In addition to prior drug exposure, agents were selected based on the results of the molecular interrogation of the tumor cells as well as the mechanisms of action and pathway interactions of the agents in order to maximize potential effectiveness and minimize overlapping toxicity. Evidence in the literature regarding effectiveness of combinations of agents identified from the molecular analysis was used to prioritize agent selection. This patient received treatment with a combination of gemcitabine and docetaxel as bridging chemotherapy while awaiting recommendations from the MTB. Her report indicated that her tumor was sensitive to gemcitabine. Due to its long track record of being used to treat pediatric solid tumors successfully and because her tumor appeared to be sensitive to gemcitabine (Z-score = 3.3 from the tumor analysis), it was decided to continue gemcitabine as part of her treatment plan going forward. Of note, this analysis predicted her tumor to be highly resistant to Taxels (and therefore docetaxel was discontinued) as well as anthracyclines, and platinum-based drugs.

In this specific tumor analysis, the mTOR inhibitor class of agents (via targeting AKT1 overexpression) scored the highest when compared to normal references with a Z-score of 3.7 and scored in the 96 percentile when compared to Cancer Reference Control (CRC). mTOR inhibitors have been tested in pediatric clinical trials, either alone or in combination with a number of other agents including some found on the subject’s report. Many of these combinations were reported to be well-tolerated. In a Phase I study in adult patients with solid tumors, the combination of everolimus, gemcitabine ± cisplatin was found to be well-tolerated, with all the dose limiting toxicities (DLTs) being hematologic, specifically thrombocytopenia (Costello et al., 2014). Thus, a recommendation was made for the mTOR inhibitor, everolimus, to be included in the drug treatment plan for this patient. Since mTOR inhibitors can be chemo-sensitizing, the patient was closely observed for toxicity with the plan to dose reduce the other agents when necessary.

Targets of dasatinib were found to be highly overexpressed in this patient’s tumor. These included CSF1R (Z-score = 3.3) and EPHA2 (Z-score = 2.2) as well as SRC (Z-score = 1.9) and TEC (Z-score = 1.1) at very high CRC (0.99–1.0). There are a few reports in the literature of clinical activity of dasatinib in combination with gemcitabine in adults with advanced solid tumors (Hong et al., 2013). Sorafenib was considered, but its target is limited to CSF1R whereas dasatinib targets 3 other pathway nodes and with pharmacy evaluation regarding the combination, dasatinib was selected as it was expected to be less toxic than Sorafenib.

Another class of drugs that scored highly in this case was the statins via targeting of MMP9, MMP14 and ICAM1 overexpression with Z-score = 3.4; CRC = 0.94, Z-score = 2.8; CRC = 0.85 and Z-score = 1.4; CRC = 0.87 respectively, for the stain pravastatin. Matrix metalloproteinases (MMPs) is a family of zinc-containing endopeptidases that degrade various components of the extra cellular matrix (ECM) and can release various growth factors entrapped within the ECM. MMPs have been implicated in cancer cell invasion, proliferation, adhesion and migration as well as tumor angiogenesis (Kawata et al., 2001). The tumor analysis report also included doxycyline due to its ability to target MMPs (Qin et al., 2014) with potential synergy with gemcitabine. Thus, doxycycline in combination with gemcitabine, everolimus, and dasatinib was the treatment plan recommended by the MTB (diagrammed in Figure 3).

Statins inhibit MMP mRNA expression and enzymatic activity. There are a few reports of clinical trials of statins alone or in combination with chemotherapy in hepatocellular carcinoma and one report in pediatric solid tumors (Kawata et al., 2001). In addition to targeting MMPs, statins can block mTOR-mediated AKT signaling and can sensitize p53-deficient cells to chemotherapy drugs, such as etoposide, dox, and 5-FU (Roudier et al., 2006). Overall, among the statin drugs, pravastatin was favored as it is well-tolerated with minimal drug-drug interactions and offered the broadest gene targeting profile among the statins reported.

Once the patient-derived cell line was established, the drug combination of gemcitabine, everolimus, dasatinib and doxycycline was tested *in vitro*. The single agents alone showed little effect, suggesting tumor resistance to single agents, while combination treatment resulted in cell toxicity. This correlates with the clinical experience involving single agent clinical trials showing less effectiveness relative to combination trials. A review of pediatric oncology trials showed that trials of combination therapies were more successful than single agents (71% vs. 28%; *p* < 0.005) (Franshaw et al., 2019). Western blot analysis confirmed upregulation of pathway intermediates predicted by RNA analysis which were suppressed using the drugs selected. This enforces the fact that when these drugs are put in combination with one another, they are more effective at decreasing cell viability in osteosarcoma than individually.

Some of the novel agents used in our case, have been studied individually or in combination for osteosarcoma treatment. Due to its exhibited activity in preclinical models of sarcoma, a multicenter Phase 2 trial of dasatinib was conducted in patients with previously treated high grade sarcoma (SARC009). Dasatinib monotherapy had some activity in patients with undifferentiated pleomorphic sarcoma but it was inactive as a single agent in the other sarcoma subtypes (Schuetze et al., 2016). Another group demonstrated that single agent dasatinib effectively inhibited the adhesion and migration of osteosarcoma cells but could not inhibit the development of pulmonary metastases in a mouse model (Hingorani et al., 2009). A precision oncology approach using the combination of dasatinib with ceritinib, an insulin like growth factor (IGF) pathway inhibitor in a patient with osteosarcoma after demonstrating benefit of the combination *in vitro* in the patient derived cell line, showed the combination was safe, but the effect was short lived (Beck et al., 2020). A non-randomized phase 2 clinical trial conducted by the Italian Sarcoma Group (NCT01804374) of sorafenib, a multi-kinase inhibitor with everolimus, using everolimus to overcome the AKT-mTOR pathway related resistance to sorafenib showed a 6-month progression free survival of 45% (Grignani et al., 2015). Everolimus increased the antitumor effect of sorafenib by abrogating mTORC2 upregulation caused by sorafenib (Pignochino et al., 2013). Doxycycline has been shown to inhibit the progression of metastases in osteosarcoma by downregulating the expression of MMPs, VEGF and Ezrin, suggesting that reprofiling of Doxycycline can prevent the evolvement of pulmonary micro-metastases to clinically detectable macro-metastases and suppress the lethal progress of Osteosarcoma by inhibiting the expression of MMPs, VEGFA and ezrin at primary sites (Hadjimichael et al., 2022; Fife et al., 1997).

Together, these results suggest that while these agents individually have activity against Osteosarcoma, their effect is short lived as a single agent. Combination treatment based on the patient’s tumor profiling offers the opportunity to target multiple pathways and overcome drug resistance as demonstrated by our patient’s lasting exceptional response.

## Conclusion

Combination treatment with molecular guided therapy determined by a molecular tumor board resulted in a clinical response as well as long-term survival in a highly refractory osteosarcoma patient. In addition, this therapeutic approach was found to be safe without significant adverse events. The osteosarcoma cell line established from the patient’s tumor confirmed improved response to combination treatments relative to single agent treatment *in vitro*.

## Data Availability

The datasets presented in this study can be found in online repositories. The names of the repository/repositories and accession number(s) can be found below: https://www.ncbi.nlm.nih.gov/gap/, phs002238.v1.
